# Gastric cancer and image-derived quantitative parameters: Part 2—a critical review of DCE-MRI and ^18^F-FDG PET/CT findings

**DOI:** 10.1007/s00330-019-06370-x

**Published:** 2019-08-07

**Authors:** Lei Tang, Xue-Juan Wang, Hideo Baba, Francesco Giganti

**Affiliations:** 1 Key Laboratory of Carcinogenesis and Translational Research (Ministry of Education), Department of Radiology, Peking University Cancer Hospital, Beijing, China; 2 Key Laboratory of Carcinogenesis and Translational Research (Ministry of Education), Department of Nuclear Medicine, Peking University Cancer Hospital, Beijing, China; 3 Department of Gastroenterological Surgery, Graduate School of Medical Sciences, Kumamoto University, Kumamoto, Japan; 4 Department of Radiology, University College London Hospital NHS Foundation Trust, London, UK; 5 Division of Surgery and Interventional Science, Faculty of Medical Sciences, University College London, 3rd Floor, Charles Bell House, 43-45 Foley Street, London, W1W 7TS UK

**Keywords:** Stomach neoplasms, Biomarkers, Magnetic resonance imaging, Positron emission tomography, Quantitative parameters

## Abstract

**Abstract:**

There is yet no consensus on the application of functional imaging and qualitative image interpretation in the management of gastric cancer. In this second part, we will discuss the role of image-derived quantitative parameters from dynamic contrast-enhanced magnetic resonance imaging (DCE-MRI) and ^18^F-fluorodeoxyglucose positron emission tomography/computed tomography (^18^F-FDG PET/CT) in gastric cancer, as both techniques have been shown to be promising and useful tools in the clinical decision making of this disease. We will focus on different aspects including aggressiveness assessment, staging and Lauren type discrimination, prognosis prediction and response evaluation. Although both the number of articles and the patients enrolled in the studies were rather small, there is evidence that quantitative parameters from DCE-MRI such as K^trans^, V_e_, K_ep_ and AUC could be promising image-derived surrogate parameters for the management of gastric cancer. Data from ^18^F-FDG PET/CT studies showed that standardised uptake value (SUV) is significantly associated with the aggressiveness, treatment response and prognosis of this disease. Along with the results from diffusion-weighted MRI and contrast-enhanced multidetector computed tomography presented in Part 1 of this critical review, there are additional image-derived quantitative parameters from DCE-MRI and ^18^F-FDG PET/CT that hold promise as effective tools in the diagnostic pathway of gastric cancer.

**Key Points:**

• *Quantitative analysis from DCE-MRI and*^*18*^*F-FDG PET/CT allows the extrapolation of multiple image-derived parameters.*

• *Data from DCE-MRI (K*^*trans*^*, V*_*e*_*, K*_*ep*_* and AUC) and *^*18*^*F-FDG PET/CT (SUV) are non-invasive, quantitative image-derived parameters that hold promise in the evaluation of the aggressiveness, treatment response and prognosis of gastric cancer.*

## Introduction

Gastric cancer (GC) is one of the most common malignancies worldwide [1]. As already discussed in the first part (Part 1) of this critical review [2], this disease is managed through a standardised multidisciplinary approach where radiology plays a crucial role in the detection, staging, treatment planning and follow-up [3, 4].

The most useful techniques are endoscopic ultrasound, computed tomography (CT), magnetic resonance imaging (MRI) and ^18^F-fluorodeoxyglucose positron emission tomography (^18^F-FDG PET)/CT. At this regard, the PLASTIC trial [5] is an ongoing study that will evaluate the impact and cost-effectiveness of PET and staging laparoscopy in addition to initial staging in patients with locally advanced GC.

Different image-derived quantitative parameters from these techniques could be considered promising tools in the management of GC [6, 7], as they reflect a variety of biological processes (normal or pathological) both at baseline and after therapeutic interventions.

Quantitative imaging has the potential to improve the value of diagnostic testing and enhance clinical productivity and is increasingly important in preclinical studies, clinical research, and clinical practice [7]. Oncological imaging represents an ideal setting for the collection of new image-derived quantitative parameters from different techniques that can be potentially included in the clinical scenario [6]. The Radiological Society of North America underlined their importance as non-invasive tools with different applications in oncology and has promoted their use in clinical trials [7].

In the second part, we will provide a critical review on the state of the art of dynamic contrast-enhanced (DCE) MRI and ^18^F-FDG PET/CT findings.

## Evidence acquisition

**Fig. 1:**
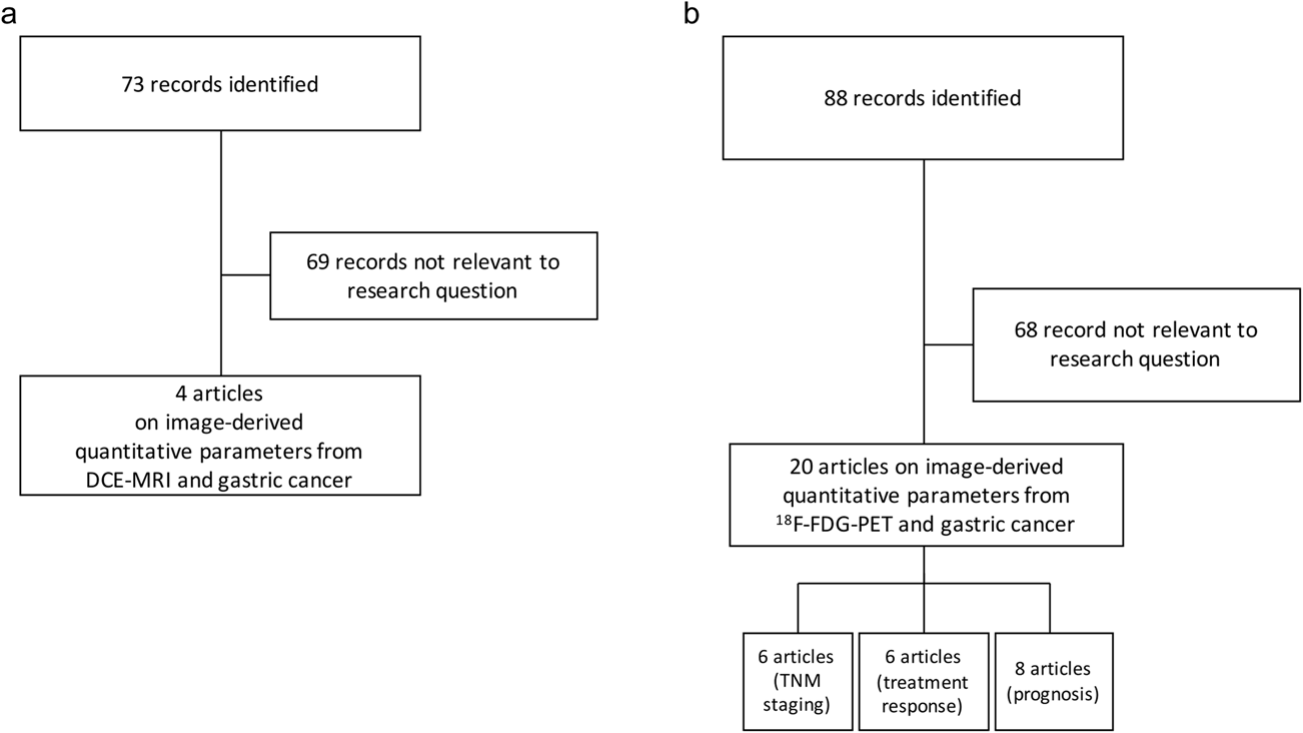
Flow diagrams showing the outcome of the initial searches resulting in the full studies included in the review for dynamic contrast-enhanced magnetic resonance imaging (DCE-MRI) (**a**) and ^18^F-fluorodeoxyglucose positron emission tomography/computed tomography (^18^F-FDG PET/CT) (**b**)

We searched MEDLINE/PubMed for manuscripts published from inception to 17 August 2018 (Fig. 1).

## DCE-MRI and image-derived quantitative parameters

DCE-MRI is a functional imaging technique in which multiphase images are acquired over a few minutes at baseline, during and after rapid intravenous injection of a contrast agent and a saline flush. Changes in signal intensity (reflecting tissue vascularity) can be observed and parametric maps of specific microvascular image-derived quantitative parameters can be derived [8, 9]. Basic recommendations include an adequate spatial/temporal resolution and knowledge of the inherent characteristics of the contrast agent. Semi-quantitative and quantitative analysis can be performed on specific regions of interest (ROIs) or on a pixel-by-pixel basis.

DCE-MRI requires high temporal resolution (usually 4–6 s/phase) and can be degraded by motion artefacts (e.g. respiratory or bowel peristalsis) [10]. Therefore, an injection of intravenous/intramuscular anti-peristaltic agent is advised to reduce the mobility of the gastric walls.

DCE-MRI reflects tumour angiogenesis (i.e. the creation of new blood vessels) and is directly associated with tumour growth and inversely correlated with prognosis [11–13].

Different quantitative parameters can be extrapolated from DCE-MRI maps (Tofts model) [14] such as:

- K^trans^ (min^−1^): volume transfer constant of gadolinium from blood plasma to the extravascular extracellular space (EES)
- V_e_ (0 to 100%): volume of the EES per unit volume of tissue (i.e. the amount of “space” available within the interstitium for accumulating gadolinium)
- K_ep_ (min^−1^): rate constant gadolinium reflux from the EES back into the vascular system (i.e. it is the ratio: K^trans^/V_e_)
- AUC (mmol/s): area under the gadolinium concentration curve during a certain period of time.

The application of DCE-MRI in GC has been increasingly growing over the last few years thanks to the technical developments (e.g. the shortening of temporal resolution) and the advantage of free-from-radiation damage compared with CT.

Although certainly interesting in a research context, this technique has been mainly applied for neuro-oncological imaging so far. However, DCE-MRI in organ systems outside the central nervous system for oncological applications remains an active area of research, especially for breast, liver and prostate cancer. Other applications of DCE-MRI have been investigated, but as yet are not routinely used in clinical practice for GC. A possible explanation is that tumours are biologically complex structures and, differently from other organs such as the brain, the DCE-MRI protocols for GC are flawed by the presence of several artefacts (especially due to peristalsis) that can easily undermine the quality of the scan and the interpretation of quantitative data from the regions of interest analysed.

### DCE-MRI in the detection and diagnosis of gastric cancer

Table 1 summarises the main studies analysing the role of DCE-MRI in GC.

**Table 1:** Dynamic contrast-enhanced magnetic resonance imaging (DCE-MRI) and gastric cancer

Study (ref.)	Year	Country	Type of study	No. of patients	MRI system	DCE acquisition	ROI placement	Imaging parameter	Key message
Kang et al [15]	2000	South Korea	Prospective	46	1.5 T	Precontrast 30, 60, and 90 s after injection Delayed scan 4–5 min after injection	Normal and pathologic gastric wall by 2 radiologists in consensus (single slice)	Thickness of the gastric wall Time to intensity curve (peak enhancement)	Stomach cancer has a thickened wall with rapid enhancement Pathological mucosa and/or submucosa show early enhancement pattern Dynamic and delayed MRI can predict preoperative T staging
Joo et al [16]	2014	South Korea	Prospective	27^a^	3 T	Radial VIBE sequences continuously scanned for 75 s Repeated volumetric sets of axial images at 4.1-s intervals for 308 s	Normal and pathologic gastric wall by 1 radiologist (single slice)	K^trans^ K_ep_ V_e_ iAUC (first 60 s)	V_e_ and iAUC are significantly higher in gastric cancer V_e_ is positively correlated with T staging K^trans^ is significantly correlated with EGFR expression DCE-MRI parameters provide prognostic information for gastric cancer.
Ma et al [17]	2016	China	Prospective	32	3 T	Acquisition time, 15 s Sequence was repeated 20 times at 10-s intervals	Pathologic gastric wall by 1 radiologist (single slice)	K^trans^ K_ep_ V_e_ iAUC (first 60 s)	Mucinous adenocarcinomas show higher V_e_ and lower K^trans^. Diffuse type shows higher V_e_ than the intestinal type Mean K^trans^ is positively correlated with VEGF DCE-MRI predicts tumour histological type, Lauren classification and estimation of tumour angiogenesis
Li et al [18]	2017	China	Prospective	43^b^	3 T	Total acquisition time = 4 min 26 s (FB radial-VIBE) + 20 s for conventional BH VIBE	Normal and pathologic gastric wall by 1 radiologist (single slice)	K^trans^ K_ep_ V_e_ iAUC (first 60 s)	Gastric cancer shows higher V_e_ and lower K_ep_

*MRI* magnetic resonance imaging, *DCE* dynamic contrast-enhanced, *ROI* region of interest, *s* seconds, *VIBE* volume-interpolated breath-hold examination, *K*^*trans*^ volume transfer coefficient, *K*_*ep*_ reverse reflux rate constant, *V*_*e*_ extracellular extravascular volume fraction, *iAUC* initial area under the gadolinium concentration curve, *EGFR* epidermal growth factor receptor, *FB* free-breathing, *BH* breath-hold

^a^But 22 with DCE-MRI of diagnostic quality

^b^But perfusion analysis on 40 patients

The first study by Kang and colleagues dates back to 2000 [15] and reports the usefulness of dynamic and delayed MRI for T staging. The thickness and enhancement pattern of normal and pathological gastric walls were compared in 46 patients through a dynamic protocol including precontrast images and additional acquisitions of 30, 60, 90 and 240–300 s after injection of gadolinium. The pathological outer layers (mucosa and submucosa) showed earlier enhancement (i.e. between 30 and 90 s) than the normal gastric wall in 43/46 patients (93%) and the peak enhancement of the normal gastric wall was > 90 s in 17/46 patients (37%). A reasonable high consistency between MR staging and pathological staging for all T stages was reported (accuracy for T stage, 83%). Such results, although not related to any specific quantitative parameter, show that dynamic MR imaging was already a promising technique for predicting T staging in GC at that time.

Joo and colleagues [16] correlated DCE-MRI parameters with prognostic factors such as pathological T staging and epidermal growth factor receptor (EGFR) expression. V_e_ and iAUC were significantly higher for GC (0.133 and 5.533 mmol/s, respectively) when compared with normal gastric wall (0.063 and 3.894, respectively) (all *p* < 0.05). Additionally, V_e_ was positively correlated with T staging (*ρ* = 0.483, *p* = 0.023) and K^trans^ was significantly correlated with EGFR expression (*ρ* = 0.460, *p* = 0.031). These findings suggest that DCE-MRI reflects tumour biology, providing prognostic information in patients with GC.

Ma and colleagues [17] compared DCE-MRI parameters in different histological subtypes of GC and investigated their correlation with vascular endothelial growth factor (VEGF) expression levels in 32 patients treated with surgical resection. Differently from the other studies, the ROIs were placed only on the lesions and the size was constant for each patient (10 mm). Mucinous adenocarcinomas showed higher V_e_ (0.491) and lower K^trans^ (0.077 min^−1^) values than non-mucinous tumours (0.288 and 0.274 min^−1^, respectively) (*p* < 0.01). Differences were also observed for the Lauren classification, as the diffuse type showed higher V_e_ and K^trans^ (0.466 and 0.249 min^−1^, respectively) values than the intestinal type (0.253 and 0.183 min^−1^, respectively) (*p* < 0.001). Additionally, K^trans^ showed a significant correlation with the level of VEGF expression (*ρ* = 0.762, *p* < 0.001). K^trans^ and VEGF are both related to the endothelial and microvascular permeability, which are in turn related to the neo-angiogenesis that is seen in tumours: in other words, a higher K^trans^ is related to a higher level of VEGF, which is strictly related to a greater degree of angiogenesis. Together with the previous study [16], these findings suggest that angiogenesis increases the extravasation of gadolinium from the intravascular to the interstitial space, supporting the role of DCE-MRI as a potential tool to differentiate GC according to different histopathological features.

Li and colleagues [18] compared the performance of conventional breath-hold to free-breathing DCE-MRI using volume-interpolated breath-hold examination sequences. DCE-MRI parameters of normal gastric wall and GC were collected and perfusion parameters for both normal and pathological gastric walls were obtained. K_ep_ was lower (0.750 vs 1.081 min^−1^; *p* < 0.05) while V_e_ was higher in GC (0.228 vs 0.162; *p* < 0.05). No significant differences for K^trans^ and iAUC values between normal and pathological gastric walls were observed (*p* > 0.05).

**Fig. 2:**
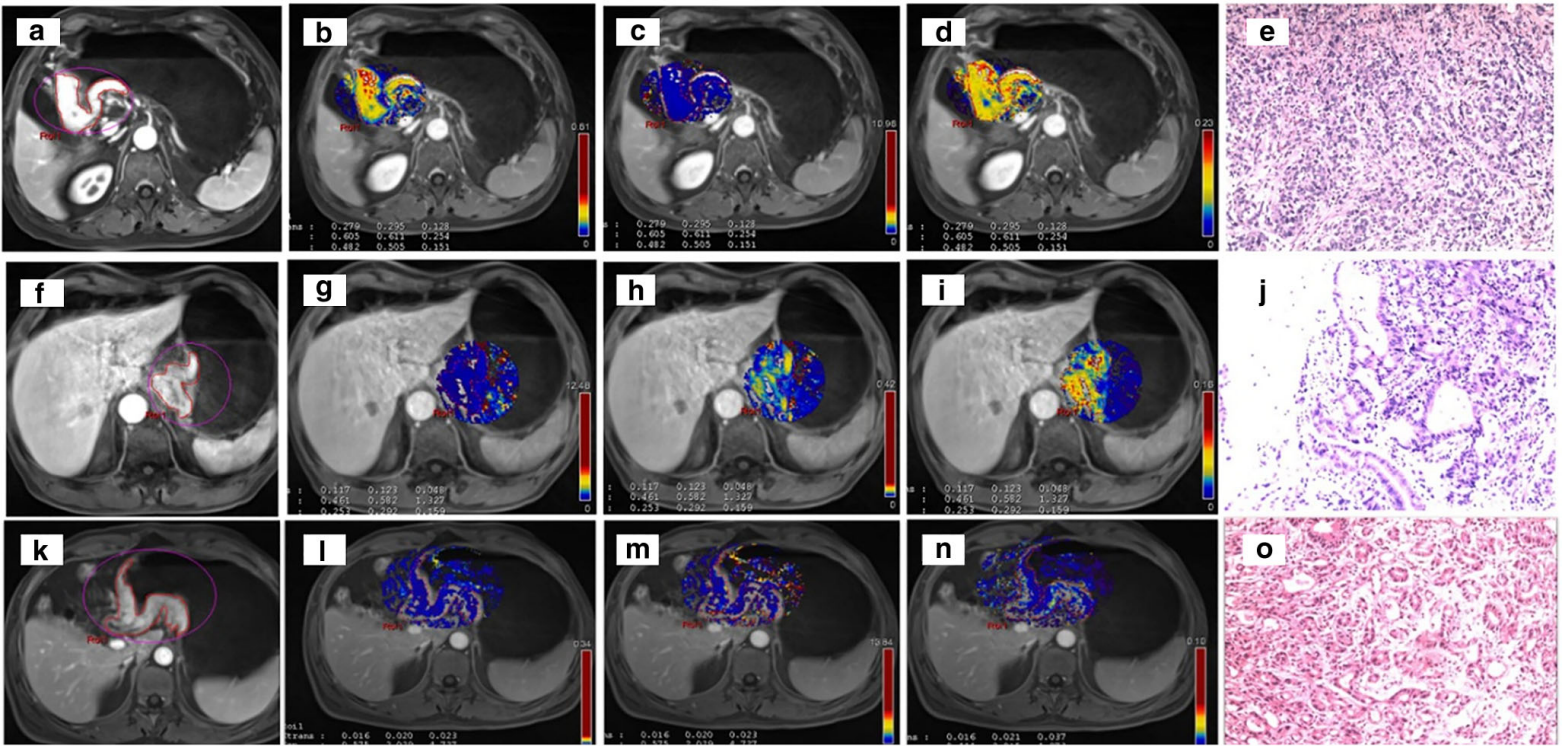
DCE-MRI showing a tumour of the gastric antrum (**a**) in a 73-year-old male. The K^trans^ (**b**) was 0.279 min^−1^, the K_ep_ (**c**) was 0.605 min^−1^ and the V_e_ (**d**) was 0.482. Final pathology (**e**): diffuse type (Lauren classification), staged as pT4aN3. DCE-MRI of a tumour of the gastro-oesophageal junction (Siewert III) (**f**) in a 68-year-old male. The K^trans^ (**g**) was 0.117 min^−1^, the K_ep_ (**h**) was 0.461 min^−1^ and the V_e_ (**i**) was 0.253. Final pathology (**j**): mixed type (Lauren classification), staged as pT3N1. DCE-MRI of a tumour of the gastric antrum (**k**) in a 49-year-old male. The K^trans^ (**l**) was 0.016 min^−1^, the K_ep_ (**m**) was 0.575 min^−1^ and the V_e_ (**n**) was 0.029. Final pathology (**o**): intestinal type (Lauren classification), staged as pT4aN2

**Fig. 3:**
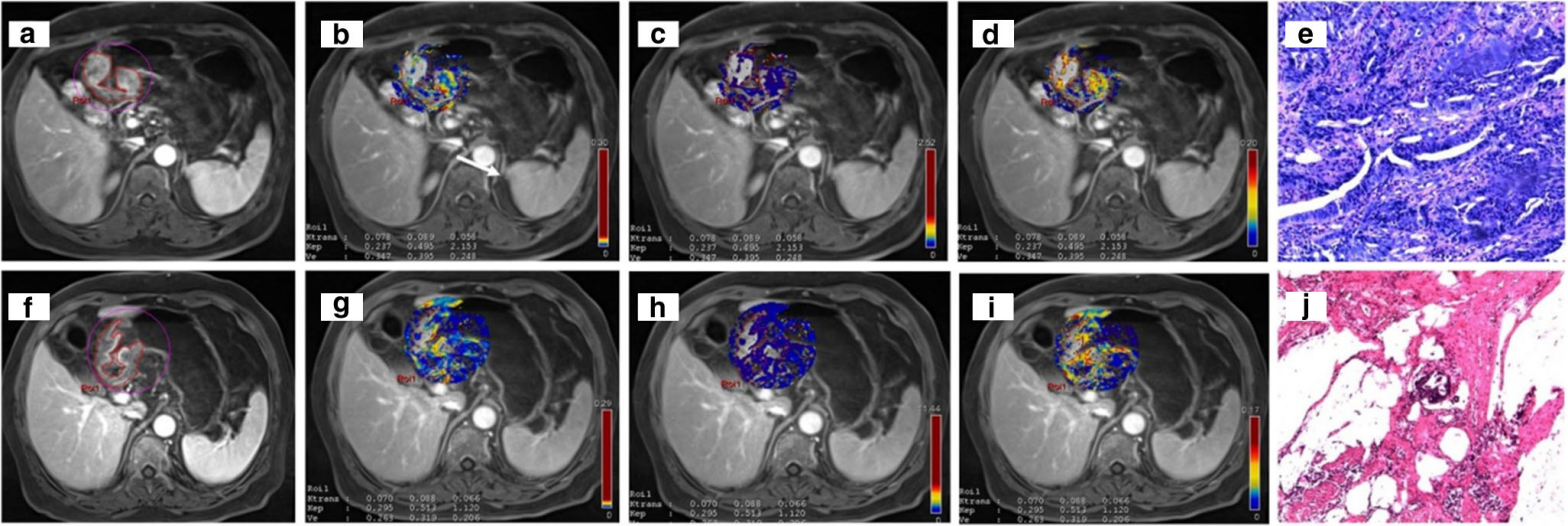
DCE-MRI showing a tumour of the gastric antrum (**a**) in a 66-year-old female. In the pretreatment scan, the K^trans^ (**b**) was 0.078 min^−1^, the K_ep_ (**c**) was 0.237 min^−1^ and the V_e_ (**d**) was 0.347. The tumour was confirmed at biopsy (**e**). In the posttreatment scan, there was a reduction in tumour size (**f**), and the K^trans^ (**g**) was 0.070 min^−1^, the K_ep_ (**h**) was 0.295 min^−1^ and the V_e_ (**i**) was 0.263. Final pathology (**j**): intestinal type (Lauren classification), staged as ypT1bN0 (tumour regression grade 1)

**Fig. 4:**
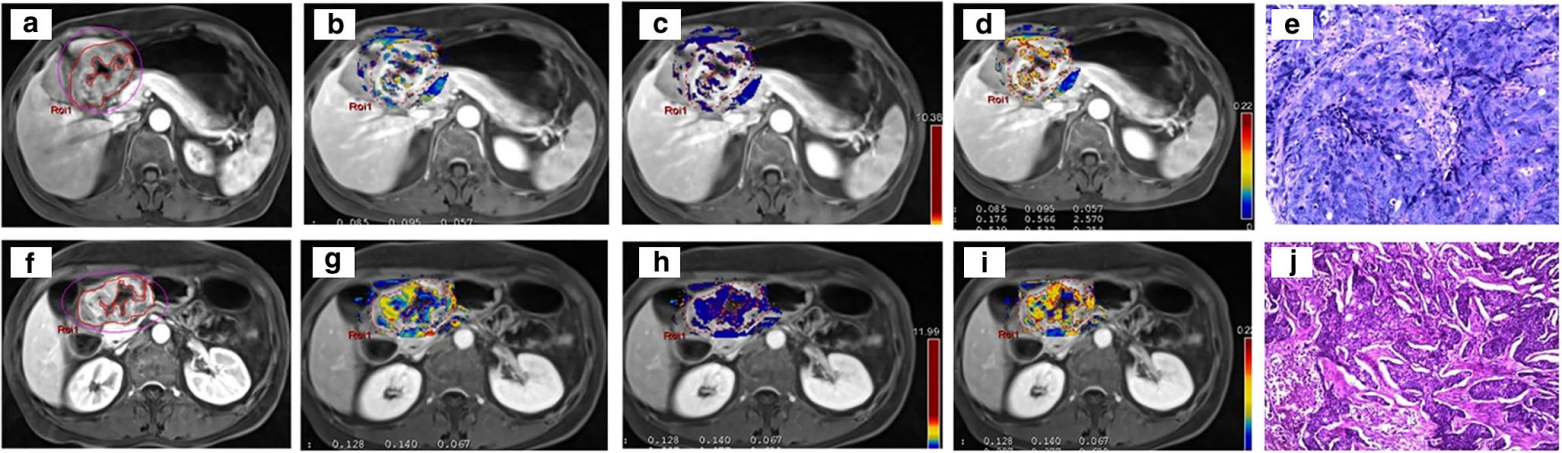
DCE-MRI of a tumour of the gastric antrum (**a**) in a 61-year-old female. In the pretreatment scan, the K^trans^ (**b**) was 0.085 min^−1^, the K_ep_ (**c**) was 0.176 min^−1^ and the V_e_ (**d**) was 0.539. The tumour was confirmed at biopsy (**e**). In the posttreatment scan, the tumour is still visible (**f**), and the K^trans^ (**g**) was 0.128 min^−1^, the K_ep_ (**h**) was 0.297 min^−1^ and the V_e_ (**i**) was 0.455. Final pathology (**j**): diffuse type (Lauren classification), staged as ypT3N0 (tumour regression grade 3)

Some examples of DCE-MRI in GC are shown in Figs. 2, 3 and 4.

## ^18^F-FDG PET/CT and image-derived quantitative parameters

^18^F-FDG PET/CT is recommended for patients with newly diagnosed GC if clinically indicated and if metastatic cancer is not evident, as well as in the posttreatment assessment and restaging.

**Fig. 5:**
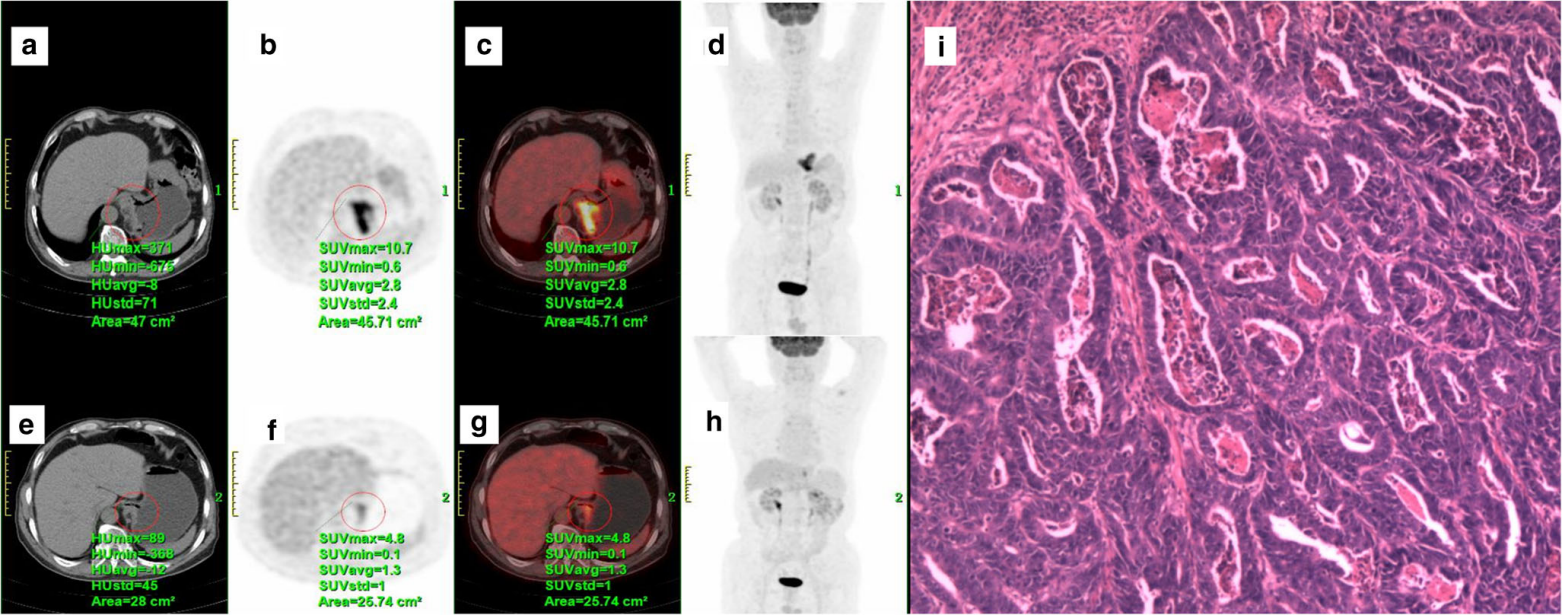
^18^F-FDG PET/CT scan of a 72-year-old man with gastro-oesophageal junction cancer (**a**–**d**) demonstrated by an intense uptake of ^18^F-FDG before treatment (SUV_max_ = 10.7) (**c**). After two cycles of chemotherapy (paclitaxel + cisplatin + fluorouracil) (**e**–**h**), the SUV_max_ of the lesion decreased to 4.8 (**g**), showing good response to the therapy. Final pathology (**i**) ypT3N0 (tumour regression grade 1)

**Fig. 6:**
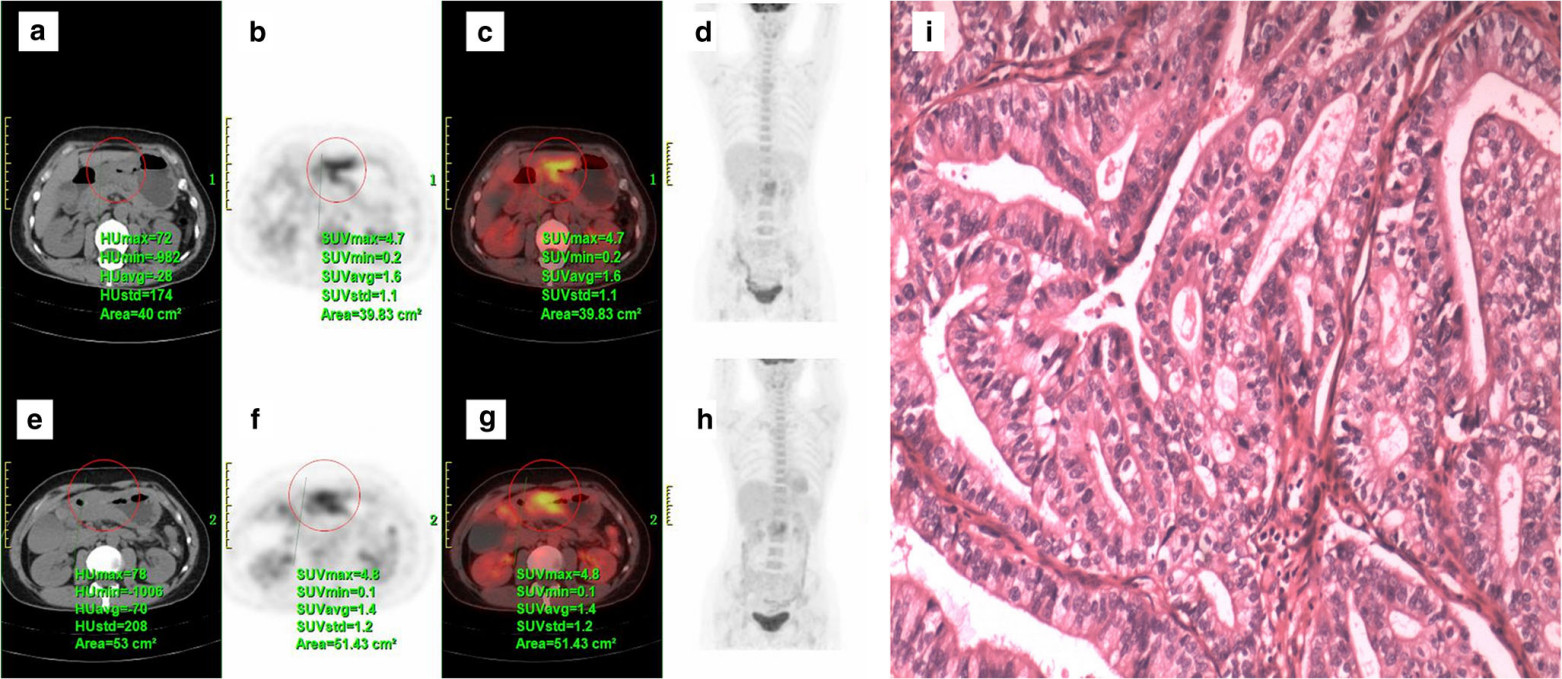
^18^F-FDG PET/CT scan of a 48-year-old woman with gastric cancer (**a**–**d**) demonstrated by an intense uptake of ^18^F-FDG before treatment (SUV_max_ = 4.7) (**c**). After one cycle of chemotherapy (capecitabine + paclitaxel) (**e**–**h**), no significant changes in ^18^F-FDG uptake (SUV_max_ = 4.8) were observed (**g**). Final pathology (**i**) ypT4aN1 (tumour regression grade 3)

The standardised uptake value (SUV) from ^18^F-FDG PET/CT is a dimensionless ratio used to distinguish between normal and abnormal levels of glucose uptake and can be considered an image-derived semi-quantitative parameter, defined as the ratio activity per unit volume of a ROI to the activity per unit whole-body volume (Figs. 5 and 6) [19].

### ^18^F-FDG PET/CT to assess the primary lesion in gastric cancer

Table 2 summarises the studies on the role of ^18^F-FDG PET/CT to assess the primary lesion in GC.

**Table 2:** ^18^F-Fluorodeoxyglucose positron emission tomography (^18^F-FDG PET) and aggressiveness in gastric cancer

Study (ref.)	Year	Country	Type of study	No. of patients	ROI placement	SUV cut-off	Reference standard	Key messages
Stahl et al [20]	2002	Germany	Prospective	40 (+ 10 controls)	Tumour and normal gastric wall	4.6	Biopsy	^18^F-FDG PET detected 24/40 (60%) of locally advanced gastric cancers The mean SUV was higher in the intestinal type than in the non-intestinal type (6.7 vs 4.8; *p* = 0.03) The survival rate of patients (*n* = 36) with ^18^F-FDG accumulation did not differ from those with low ^18^F-FDG accumulation (*p* = 0.75)
Mochiki et al [21]	2004	Japan	Prospective	156	Tumour, lymph nodes and normal gastric wall	4	Radical surgery	Significant association between SUV and the tumour invasion, size and nodal metastasis ^18^F-FDG PET is less accurate than CT in nodal staging (sensitivity, 23% vs. 65%, respectively) Survival rate for SUV > 4 was lower than for SUV < 4 (*p* < 0.05) ^18^F-FDG PET is not feasible for detecting early-stage gastric cancers
Chen et al [22]	2005	South Korea	Prospective	68	Tumour	Three-point scale: 1 (normal), 2 (equivocal) and 3 (abnormal)^a^	Radical surgery	^18^F-FDG PET sensitivity was 94% in patients with gastric cancer Significant association between ^18^F-FDG uptake and tumour size, nodal involvement and other histological features ^18^F-FDG PET + CT is more accurate for preoperative staging than either modality alone (66% vs. 51% and 66% vs. 47%; *p* = 0.002)
Oh et al [23]	2011	South Korea	Retrospective	136	Tumour	3.2	Radical surgery	SUV was significantly associated with tumour size, depth of invasion and nodal metastasis (*p* < 0.001) but not with tumour histology (*p* = 0.099)
Oh et al [24]	2012	South Korea	Retrospective	38	Tumour	Measurable disease was defined as 1.35*SUV_max_ of liver+2*standard deviation of liver SUV	Radical surgery	31/38 (82%) of tumours were visible on ^18^F-FDG PET Measurable tumours on ^18^F-FDG PET were more frequent in well- or moderately differentiated gastric cancer (*p* < 0.05), antrum or angle and intestinal type (*p* > =0.05)
Namikawa et al [25]	2013	Japan	Retrospective	90	NR	NR	Radical surgery	^18^F-FDG PET CT sensitivity for gastric cancer was 79% Median SUV_max_ was significantly different in patients with T3/T4 disease, distant metastasis and stage III/IV tumours The SUV_max_ was correlated with tumour size (*r* = 0.461; *p* < 0.001)

*ROI* region of interest, *SUV* standardised uptake value, *PET* positron emission tomography, *FDG* fluorodeoxyglucose, *CT* computed tomography

^a^2 and 3 were considered positive

Stahl and colleagues [20] analysed the relationship between SUV_mean_ and different tumour features from biopsy (including intestinal vs non-intestinal) in 40 patients. PET had a sensitivity of 60% in identifying locally advanced GC and the SUV_mean_ was higher in the intestinal than in the non-intestinal type (6.7 vs 4.8; *p* = 0.03). No significant differences in the survival rate of patients with or without FDG accumulation (SUV_mean_ cut-off, 4.6; *p* = 0.75) were observed. A clear limitation of this study is that the reference standard was biopsy and not radical surgery.

Mochiki and colleagues [21] reported a significant association between SUV_mean_ and the depth of invasion, tumour size and nodal metastasis. They compared ^18^F-FDG PET findings with CT and found that ^18^F-FDG PET was less accurate for nodal staging (23% vs 65%). The SUV_mean_ was higher for T2–T4 than T1 tumours (*p* < 0.05). Differently from the previous study [20], they observed a significant difference in the survival rate (*p* < 0.05).

Chen and colleagues [22] reported a sensitivity of 94% for ^18^F-FDG PET/CT (SUV_mean_ = 7) and a significant association between FDG uptake and tumour size, nodal involvement and other histological features. They were among the first showing that the combination of ^18^F-FDG PET and CT was more accurate for preoperative staging than either modality alone (66% vs 51%, 66% vs. 47%; *p* = 0.002).

Oh and colleagues [23] performed a retrospective ^18^F-FDG PET/CT analysis of 136 patients treated with radical surgery. They set a threshold for SUV_peak_ from primary tumour of 3.2 to define hypermetabolic lesions and found that this was associated with tumour depth and nodal involvement (*p* < 0.001). The sensitivity and specificity for nodal involvement using the aforementioned threshold were 75% and 74% respectively.

Another group [24] reported the relationship between measurable and non-measurable GC on ^18^F-FDG PET/CT (defined as 1.35*SUV_max_ of liver+2*standard deviation of liver SUV). Among different parameters, a higher proportion of measurable tumours was found in well- or moderately differentiated GC than poorly differentiated tumours (71% vs 33% *p* < 0.05). Differently from the previous study [24], there was no difference for primary tumour stage and nodal metastasis.

Namikawa and colleagues [25] reported a sensitivity of 79% for the detection of GC for ^18^F-FDG PET/CT and a significant difference for SUV_max_ for patients with T3/T4 vs T1/T2 (9.0 vs. 3.8; *p* < 0.001), with and without distant metastasis (9.5 vs. 7.7; *p* = 0.018), and between stage III/IV and stage I/II (9.0 vs. 4.7; *p* = 0.017) after radical surgery. The SUV_max_ of the primary tumour was correlated with tumour size (*r* = 0.461; *p* < 0.001). The sensitivity, specificity and accuracy of ^18^F-FDG PET/CT for nodal involvement were 64%, 86% and 71% respectively.

### ^18^F-FDG PET/CT in treatment response of gastric cancer

We found six studies reporting on ^18^F-FDG PET/CT and treatment response in GC (Table 3).

**Table 3:** Fluorodeoxyglucose positron emission tomography (^18^F-FDG PET) and treatment response in gastric cancer

Study (ref.)	Year	Country	Type of study	No. of patients	ROI placement	SUV reduction to distinguish between responders and non responders	Number of ^18^F-FDG PET scans	Histological definition of treatment response	Reference standard	Key messages
Stahl et al [26]	2004	Germany	Retrospective	43	Tumour	40%	Baseline and during the first cycle of chemotherapy	< 10% viable tumour cells in the specimen	Surgery	Pretreatment SUV was higher for responders than non-responders (*p* = 0.09) SUV after the first cycle of chemotherapy was lower for responders than non-responders (*p* = 0.36) SUV changes were significantly higher in responders than non-responders (*p* < 0.01) Importance of protocol standardisation
Vallböhmer et al [27]	2013	Germany	Prospective	40	Tumour	NR	Baseline and 2 weeks after completion of chemotherapy	< 10% viable tumour cells in the specimen	Surgery	Overall, posttreatment SUV was significantly lower than pretreatment SUV (*p* = 0.0006) No significant correlations between pre- and posttreatment SUV (and relative changes) and histological treatment response Higher pretreatment SUV for intestinal (7.8) than diffuse (5.1) types (*p* = 0.023) SUV change was significantly different according to tumour location (*p* = 0.041).
Giganti et al [28]	2014	Italy	Prospective	17	Tumour	NR	Baseline and 2 weeks after completion of chemotherapy	TRG 1–3 were considered responders and TRG 4–5 non-responders	Surgery	No correlations between pre- or posttreatment SUV (and % change) and treatment response
Wang et al [29]	2015	China	Prospective	64	Tumour + metastatic sites (liver, nodes and ovary)	40% (primary tumour)	Baseline and 14 days after start of chemotherapy	NR^a^	Imaging (unresectable gastric cancer)	A 40% uptake reduction is the cut-off to predict clinical response (sensitivity of 70% and specificity of 83%) to predict Early metabolic change might be a predictive marker for response and disease control in advanced gastric cancer
Park et al [30]	2016	South Korea	Prospective	74	Tumour	50%	Baseline and 6 weeks after start of chemotherapy	NR	Imaging (unresectable gastric cancer)	A 50% SUV_max_ reduction was associated with a 30% tumour size reduction (*p* < 0.001) Poorly cohesive carcinomas demonstrate lower SUV_max_ irrespective of tumour size (*p* < 0.001) HER2–positive tumours showed increased SUV_max_ than HER2–negative tumours (*p* = 0.002)
Schneider et al [31]	2018	Switzerland	Retrospective	30	Tumour	35%	Baseline and 2 weeks after the completion of chemotherapy	< 10% viable tumour cells in the specimen	Surgery	Metabolic response was observed in 67% and no response in 33% Prediction of pathological response by SUV had a sensitivity of 91% and a specificity of 47%, with an overall accuracy of 63%

*ROI* region of interest, *SUV* standardised uptake value, *PET* positron emission tomography, *NR* not reported, *TRG* tumour regression grade, *HER* human epidermal growth factor receptor

^a^RECIST criteria were used

Stahl and colleagues [26] compared different ^18^F-FDG PET/CT protocols and calculations of the SUV_mean_ (time delay after ^18^F-FDG administration, acquisition protocol, reconstruction algorithm, SUV normalisation) for the early prediction of treatment response at baseline and after the first cycle of chemotherapy. They did not find any significant difference in the baseline and follow-up SUV_mean_ calculation between protocols (*p* > 0.05), but higher SUV changes for responders than non-responders were observed (*p* < 0.01). They were among the first to demonstrate the robustness of ^18^F-FDG PET/CT for therapeutic monitoring, supporting the comparability of studies obtained with different protocols.

Vallböhmer and colleagues [27] analysed the differences in pre- and posttreatment SUV_max_ between responders and non-responders using the same histological definition as Stahl [26] (i.e. < 10% viable tumour cells in the specimen) but no correlation with treatment response was observed (*p* = 0.733). Significant differences in SUV_max_ were observed for the Lauren classification (*p* = 0.023) and tumour location (*p* = 0.041).

In another study on 17 patients [28] undergoing diffusion-weighted MRI and ^18^F-FDG PET/CT before and after treatment, no differences in treatment response were observed for pre- or posttreatment SUV_mean_ (and their percentage change) (*p* = 0.605, *p* = 0.524 and *p* = 0.480). Treatment response was based on tumour regression grade (TRG) [32] and responders were considered TRG 1, 2 and 3 (i.e. including patients with more than 10% of viable cells).

Two studies [29, 30] evaluated the relationship between SUV_max_ and treatment response in advanced GC (i.e. no surgical specimens were used as the reference standard). Although follow-up imaging was performed at different time points (14 days vs 6 weeks after the start of chemotherapy) and different SUV thresholds for response were applied (40% vs 50%), both studies showed that metabolic changes in ^18^F-FDG PET/CT are predictive markers for response disease also for advanced GC. One study [30] showed a correlation between human epidermal growth factor HER2 status positivity (i.e. more aggressive cancer) and higher SUV uptake (*p* = 0.002).

Schneider and colleagues [31] reported that ^18^F-FDG PET/CT is able to detect non-responders (sensitivity, 91%; specificity, 47%; positive predictive value, 50%; negative predictive value, 90%; accuracy, 63%) but they could not prove that ^18^F-FDG PET/CT after the first cycle of chemotherapy can predict overall pathological response.

Similarly to the PRIDE study in oesophageal cancer [33], there is growing interest to develop models that predict the probability of response to neoadjuvant therapy in GC based on quantitative parameters derived from MRI and ^18^F-FDG PET/CT. However, given the controversial results at this regard [34], further studies are needed.

### ^18^F-FDG PET/CT in the prognosis of gastric cancer

We found eight studies on ^18^F-FDG PET/CT and prognosis in GC (Table 4). Significant results on the relationship between SUV_max_ and SUV_mean_ and overall survival were reported by seven of them [35–38, 40–42], even though each study used different SUV_max_ and SUV _mean_ cut-offs (Table 4). The study that did not show any significant difference in SUV_max_ and SUV_mean_ with regard to prognosis was performed by Grabinska and colleagues [39]. A possible explanation is that a long range of follow-up was introduced in this study (range, 6 days to 5.2 years; median, 9.5 months), as also reported by the same authors. Therefore, the survival analysis from their study should be interpreted with caution. However, there is evidence of the relationship between SUV_max_ and SUV_mean_ and prognosis in GC (Table 4).

**Table 4:** ^18^F-Fluorodeoxyglucose positron emission tomography (^18^F-FDG PET) and prognosis in gastric cancer

Study (ref.)	Year	Country	Type of study	No. of patients	Follow-up (months)	ROI placement	SUV cut-off for stomach	Reference standard	Key message
Pak et al [35]	2011	South Korea	NR	41	31	Tumour	3.80	Surgery	The high-SUV group showed more aggressive tumour behaviour in relation to TNM stages (*p* = 0.018) and more postoperative recurrence (*p* = 0.028), shorter relapse-free survival (*p* = 0.004), and lower 30-month cancer-specific survival rates (40% vs. 69.3%; *p* = 0.008) SUV is not an independent predictor of overall survival at multivariate analysis
Park et al [36]	2012	South Korea	NR	82	NR	Tumour, lymph nodes and other metastatic sites	6	Biopsy	Longer median progression-free survival (8.7 vs. 4.8 months; *p* = 0.001) and overall survival (15.4 vs. 11.2 months; *p* = 0.006) were observed for patients with SUV < 6 Among patients with histologically undifferentiated carcinomas, those with SUV < 6 showed longer median progression-survival (*p* = 0.005) and overall survival (*p* < 0.001) SUV was as an independent predictor of progression-free survival (*p* = 0.002) and overall survival (*p* = 0.038)
Lee et al [37]	2012	South Korea	Retrospective	271	24	Tumour	8.2	Surgery	Tumour size, depth of invasion, nodal involvement, positive ^18^F-FDG uptake and SUV were significantly associated with tumour recurrence at univariate analysis (*p* ≤ 0.001) Depth of invasion, positive ^18^F-FDG uptake and SUV were significantly different at multivariate analysis (*p* < 0.005) The 24-month recurrence-free survival rate was significantly higher in patients with a negative than in those with a positive ^18^F-FDG uptake (95% vs 74%; *p* < 0.0001)
Kim et al [38]	2014	South Korea	Retrospective	97	30	Tumour	5.74	Surgery	Progression-free survival of the group with SUV ≤ 5.74 was significantly longer (30.9 months) than that with SUV > 5.74 (24.3 months) (*p* = 0.008) In multivariate analysis, high SUV (> 5.74) is the only poor prognostic factor for progression-free survival (*p* = 0.002; HR = 11.03)
Grabinska et al [39]	2015	Poland	Retrospective	40	9.5	Tumour	NR for prognosis	Biopsy	Despite a difference in median SUV between confined and disseminated gastric cancer (10.36 vs 12.78), no significant difference in SUV was observed with regard to prognosis
Na et al [40]	2016	South Korea	Retrospective	133	43	Tumour	4.3	Surgery	Patients with higher SUV had shorter overall survival (*p* = 0.008) at univariate analysis but not after adjusting for other clinical parameters (*p* = 0.28) SUV was significantly associated with shorter recurrence-free survival (*p* = 0.003), but not after adjusting for other clinical factors (*p* = 0.06)
Lee et al [41]	2017	South Korea	Retrospective	44	44	Tumour	1.45^a^	Biopsy/surgery	The overall survival for patients with SUV > 1.45 was not significantly different (*p* = 0.068) at univariate analysis but it was at multivariate analysis (HR, 2.026; *p* = 0.054) The progression-free survival for patients with SUV > 1.45 was significantly different both at univariate (*p* = 0.046) and multivariate analyses (HR, 2.105; *p* = 0.036)
Chon et al [42]	2018	South Korea	Retrospective	727	32.5	Tumour	7.6^b^ 4.6^c^ 5.6^d^	Surgery	In multivariate analysis, high SUV was negatively correlated with disease-free survival (HR, 2.17) and overall survival (HR, 2.47) (both *p* < 0.001) in patients with diffuse type In multivariate analysis, high SUV was negatively correlated with disease-free survival (HR, 2.26; *p* = 0.005) and overall survival (HR, 2.61; *p* = 0.003) in patients with signet ring cell carcinoma This negative prognostic impact was not observed in patients with intestinal type or well- or moderately differentiated histology

*ROI* region of interest, *NR* not reported, *SUV* standardised uptake value, *TNM* tumour node metastasis, ^*18*^*F-FDG* 18-fluorodeoxyglucose, *HR* hazard ratio

^a^After chemotherapy

^b^Intestinal type

^c^Diffuse type

^d^Mixed type

### ^18^F-FDG PET/CT and radiomics in gastric cancer

There is growing evidence of the importance of radiomics in medical imaging [43] and this applies also to ^18^F-FDG PET/CT findings [44, 45].

A recent review has shown the promising role of radiomics obtained from different techniques—including ^18^F-FDG PET/CT—in gastro-oesophageal tumours [46].

Jiang and colleagues [47] have also developed a dedicated radiomic score using the features from ^18^F-FDG PET/CT in GC. In their study, they concluded that the radiomic signature was a powerful predictor of overall and disease-free survival and could add prognostic value to the traditional staging system.

However, as the current literature on this specific topic is still preliminary, there is a need of standardisation and different multicentre studies before including radiomics from ^18^F-FDG PET/CT in the clinical routine for GC.

## Limitations

Quantitative imaging is becoming an increasingly common tool in modern radiology and its potential impact on patient care and on clinical outcomes is huge. However, it is broadly accepted that surrogate quantitative parameters of tumour biology assessed by imaging still require extensive standardisation and validation to proof that the surrogate represents the pathophysiological process under investigation. As reported by Rosenkrantz and colleagues [48], there are some practical aspects that should be considered when discussing the role of image-derived quantitative parameters. These are (i) accuracy (of a measurement, for example); (ii) repeatability and (iii) reproducibility (especially when quantitative imaging is performed in serial scans over time, as this allows to discriminate measurement error from biologic change) and (iv) clinical validity (i.e. impacting and improving patient’s life).

Therefore, some limitations from the papers discussed in this study should be reported. Firstly, for DCE-MRI, our review shows that the ROIs in all studies have been drawn on one selected axial section. This represents an important limitation, as these findings may be less representative of the whole tumour. Future studies should perform quantitative analysis on the whole volume obtained by contouring the tumour borders on each slice by planimetry. There is also a lack of optimised perfusion MRI protocols, dedicated postprocessing software programmes and high variability between MR scanners.

As far as ^18^F-FDG PET/CT imaging is concerned, a clear limitation is that the SUV is dependent on many factors including the ROI delineation, the activity injected, plasma glucose levels, and body size. There is variability between ^18^F-FDG PET/CT scanners, as well as in the accuracy of the image reconstruction and correction algorithms. The increased ^18^F-FDG uptake can be also seen in inflammatory or granulomatous processes and in sites of physiological tracer biodistribution.

Gastric distention, achieved by the consumption of water, milk or foaming agents before scanning, and a late-time-point ^18^F-FDG PET/CT scanning can relatively differentiate the physiological uptake from the malignant lesion.

Finally, standardised guidelines on how to interpret the quantitative results from DCE-MRI and ^18^F-FDG PET/CT have yet to be reported.

## Conclusions

Similarly to the ADC from diffusion-weighted MRI and texture analysis from CT [2], different image-derived quantitative parameters from DCE-MRI and ^18^F-FDG PET/CT are promising tools in the management of GC. However, extensive standardisation and validation are still required before they can become an essential cornerstone for GC.

## Abbreviations

^18^F-FDG PET/CT: ^18^F-Fluorodeoxyglucose positron emission tomography/computed tomography
ADC: Apparent diffusion coefficient
CT: Computed tomography
DCE-MRI: Dynamic contrast-enhanced magnetic resonance imaging
EGFR: Epidermal growth factor receptor
GC: Gastric cancer
SUV: Standardised uptake value
VEGF: Vascular endothelial growth factor
HER: Human epidermal growth factor
